# Genes Associated with 2-Methylisoborneol Biosynthesis in Cyanobacteria: Isolation, Characterization, and Expression in Response to Light

**DOI:** 10.1371/journal.pone.0018665

**Published:** 2011-04-07

**Authors:** Zhongjie Wang, Yao Xu, Jihai Shao, Jie Wang, Renhui Li

**Affiliations:** 1 Key Laboratory of Aquatic Biodiversity and Conservation, Institute of Hydrobiology, Chinese Academy of Sciences, Wuhan, People's Republic of China; 2 Graduate University of Chinese Academy of Sciences, Beijing, People's Republic of China; 3 College of Geography Science, Nanjing Normal University, Nanjing, People's Republic of China; 4 Resources and Environment College, Hunan Agricultural University, Changsha, People's Republic of China; 5 College of Life Science, Shanxi University, Taiyuan, People's Republic of China; Netherlands Institute of Ecology, Netherlands

## Abstract

The volatile microbial metabolite 2-methylisoborneol (2-MIB) is a root cause of taste and odor issues in freshwater. Although current evidence suggests that 2-MIB is not toxic, this compound degrades water quality and presents problems for water treatment. To address these issues, cyanobacteria and actinomycetes, the major producers of 2-MIB, have been investigated extensively. In this study, two 2-MIB producing strains, coded as *Pseudanabaena* sp. and *Planktothricoids raciborskii*, were used in order to elucidate the genetic background, light regulation, and biochemical mechanisms of 2-MIB biosynthesis in cyanobacteria. Genome walking and PCR methods revealed that two adjacent genes, SAM-dependent methyltransferanse gene and monoterpene cyclase gene, are responsible for GPP methylation and subsequent cyclization to 2-MIB in cyanobacteria. These two genes are located in between two homologous cyclic nucleotide-binding protein genes that may be members of the Crp-Fnr regulator family. Together, this sequence of genes forms a putative operon. The synthesis of 2-MIB is similar in cyanobacteria and actinomycetes. Comparison of the gene arrangement and functional sites between cyanobacteria and other organisms revealed that gene recombination and gene transfer probably occurred during the evolution of 2-MIB-associated genes. All the microorganisms examined have a common origin of 2-MIB biosynthesis capacity, but cyanobacteria represent a unique evolutionary lineage. Gene expression analysis suggested that light is a crucial, but not the only, active regulatory factor for the transcription of 2-MIB synthesis genes. This light-regulated process is immediate and transient. This study is the first to identify the genetic background and evolution of 2-MIB biosynthesis in cyanobacteria, thus enhancing current knowledge on 2-MIB contamination of freshwater.

## Introduction

Microorganisms are an important source of terpenoid metabolites, including monoterpenes, sesquiterpenes, and diterpenes, in natural environments. Among the numerous terpenoid compounds, geosmin and 2-methylisoborneol (2-MIB) belonging to the sesquiterpenes and monoterpenes, respectively, and are widely known for their odorous and volatile properties. These two terpenoid alcohols are synthesized and usually secreted as secondary metabolites by microorganisms, such as fungi, myxobacteria, actinmycetes, and cyanobacteria [1]–[5]. Geosmin was isolated and identified as a degraded sesquiterpenoid alcohol [1], while 2-MIB was first isolated in actinomycetes as a methylated monoterpene [6]. Human taste and olfaction are sensitive to these compounds; thus 2-MIB and geosmin are regarded as major causes of the unpleasant musty or muddy taste in some freshwater [7]. Low concentrations of 2-MIB (less than 10 ng/L) could cause detectable odorous problems in drinking waters and usually leads to rejection by consumers [8].

**Figure 1:**
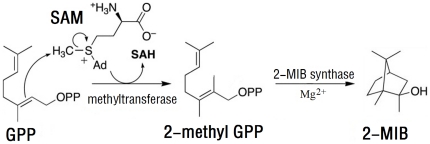
Biosynthesis pathways of 2-MIB in actinomycetes. Geranyl pyrophosohate (GPP), the universal precursor of monoterpenes, is converted to 2-MIB through two steps: methylation of GPP and cyclization of methyl-GPP subsequently.

The biochemical mechanisms of geosmin synthesis and the genetic regulation of geosmin synthesis genes have been examined in actinomycetes and cyanobacteria [9]–[11]. The universal precursor of sesquiterpenes, farnesyl diphosphate (FPP), is converted to geosmin through two steps catalyzed by a bi-functional sesquiterpene cyclase in the presence of Mg^2+^. On the other hand, 2-MIB is a methylated monoterpene alcohol, and labeling experiments have shown that the additional methyl group is transferred from S-adenosyl-L-methionine (SAM) [12]. Feeding experiments in myxobacteria conducted by Dickschat et al. [4] revealed that methylation of geranyl pyrophosohate (GPP), the universal precursor of monoterpenes, mostly occurs prior to cyclization, and that methyl-GPP is the substrate for 2-MIB cyclase. These experiments suggest that there are two steps in the biosynthesis of 2-MIB: methylation of GPP and cyclization of methyl-GPP (Figure 1). Furthermore, Komatsu et al. [13] identified genes controlling the biosynthesis of 2-MIB in the genomes of seven actinomycetes capable of producing 2-MIB. Functional analyses of these associated genes, including a monoterpene cyclase gene and a SAM-dependent methyltransferase gene, demonstrated that these genes form an operon and are responsible for the transformation of GPP to 2-MIB. Wang and Cane [14] also identified the 2-MIB cyclase gene *sco7700* and the methyltransferase gene *sco7701*, and elucidated their biochemical mechanisms in the actinomyctes strain *Streptomyces coelicolor* A3(2). They found that 2-MIB cyclase genes in actinomycetes share highly conserved motifs for Mg^2+^ binding.

Cyanobacteria are a group of photoautotrophic microorganisms prevalent worldwide with a notable impact on many ecosystems. Many cyanobacterial species produce 2-MIB. Analyses of isolated cyanobacterial strains producing taste and odor in water revealed that 2-MIB-producing species are generally non-heterocystous filamentous cyanobacteria [3]. Cyanobacteria and actinomycetes are considered the main causes of sporadic episodes of muddy tasting water from aquatic ecosystems [5], [15]. With the increase in water eutrophication and blooms in many water bodies, taste and odor problems caused by 2-MIB and geosmin have been reported frequently in recent years [16], [17]. In contrast to actinomycetes, however, no in-depth studies on the biosynthesis and genetic background of 2-MIB in cyanobacteria have been conducted. Since geosmin synthase genes in actinomycetes and cyanobacteria share a high homology [9], [11], it is possible that cyanobacteria have homologous genes and similar biosynthesis pathways for 2-MIB production. However, all cyanobacteria genomes sequenced to date are not 2-MIB producers, and genes homologous to *sco7700* and *sco7701* have not been identified from cyanobacterial genomes.

In this study, two bloom-forming cyanobacteria strains, *Pseudanabaena* sp. dqh15 and *Planktothricoides raciborskii* CHAB 3331, were isolated and confirmed to be 2-MIB-producing organisms. Based on the alignment of *sco7700* and *sco7701* with their homologous genes in actinomycetes, we designed primers for the amplification of a putative 2-MIB cyclase gene and a methyltransferase gene in these two cyanobacterial strains. In order to elucidate the genetic background of 2-MIB biosynthesis in cyanobacteria, the sequences and flanking regions of these two putative 2-MIB related genes were determined using the genome walking method. In addition, transcriptional variations of these two linked genes in response to different light intensities were examined.

## Results

### Isolation of two 2-MIB-producing cyanobacteria strains

Two non-heterocystous filamentous cyanobacterial strains exuding an “earthy” smell were obtained from water samples collected from Lake Dongqianhu, Ningbo, and Lake Donghu, Wuhan, China. Based on morphological features, one strain was identified as *Pseudanabaena* sp. and the other as *Planktothricoids raciborskii* according to the description of Komàrek and Anagnostidis [18], and coded as *Pseudanabaena* sp. dqh15 and *Planktothricoids raciborskii* CHAB 3331, respectively. The 16S rRNA genes of these strains were amplified and sequenced, and phylogenetic analysis confirmed the results of morphological identification (unpublished data).

**Figure 2:**
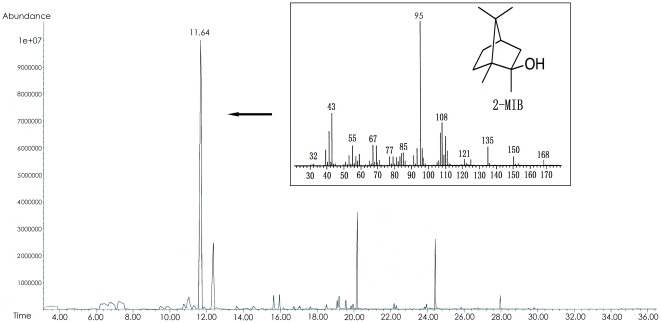
GC-MS analysis of volatile compounds obtained from the gaseous phase of the *Pseudanabaena* sp. dqh15 culture. Mass spectrum revealed that the main volatile compound (retenion time as 11.64 min, m/z 168) is 2-MIB.

Chemical analysis demonstrated that these two isolates are capable of producing 2-MIB. Using HS-SPME coupled with GC-MS, the main odorous components produced by *Pseudanabaena* sp. dqh15 and *Planktothricoides raciborskii* CHAB 3331 were isolated and determined. For *Pseudanabaena* sp. dqh15, total ion chromatography and mass spectrum revealed that the compound peak with a retention time of 11.64 min was the main odorous component, and this corresponded to 2-MIB (Figure 2). Similar results were obtained in *Planktothricoides raciborskii* CHAB 3331 (data not shown). In addition, analyses of the attached heterotrophic bacteria confirmed that 2-MIB was produced only by the cyanobacterial cells.

### Identification and characterization of 2-MIB-associated genes in the isolated strains

**Figure 3:**
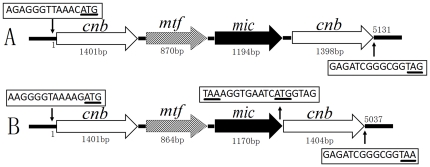
2-MIB biosynthesis associated genes identified from cyanobacterial strains. A: *Pseudanabaena* sp. dqh15; B: *Planktothricoides raciborskii* CHAB 3331. The *cnb*, *mtf* and *mic* reprents cyclic nucleotide-binding protein gene, methyltransferase gene and 2-MIB cyclase gene respectively. These genes form putative operon in chromosome.

The two genomic fragments homologous to *sco7701* were successfully amplified from both *Pseudanabaena* sp. dqh15 and *Planktothricoides raciborskii* CHAB 3331. The fragments were amplified by PCR using the primers SAMF2 and SAMR1 designed according to the sequence of the SAM-dependent methyltransferase genes in actinomycetes. The downstream and upstream regions of the known sequences were further obtained using genome walking-PCR. In *Pseudanabaena* sp. dqh15, the full length of SAM-dependent methyltransferase gene (*mtf*) was 870 bp, while a 95-bp segment downstream of *mtf* and an 1194-bp ORF was amplified and predicted to be the 2-MIB cyclase gene (*mic*). Similarly, an 864-bp *mtf* gene and a 1170-bp *mic* gene located 96-bp downstream were identified in *Planktothricoides raciborskii* CHAB 3331 (Figure 3). The *mtf* genes of *Pseudanabaena* sp. dqh15 and *Planktothricoides raciborskii* CHAB 3331 had 57.4% and 59.4% identities with *sco7701* DNA sequences, and 52.2% and 52.0% identities with the deduced amino acid sequences, respectively. For *mic* genes, the identities were 43.3% and 44.8% with *sco7700* DNA sequences.

**Figure 4:**
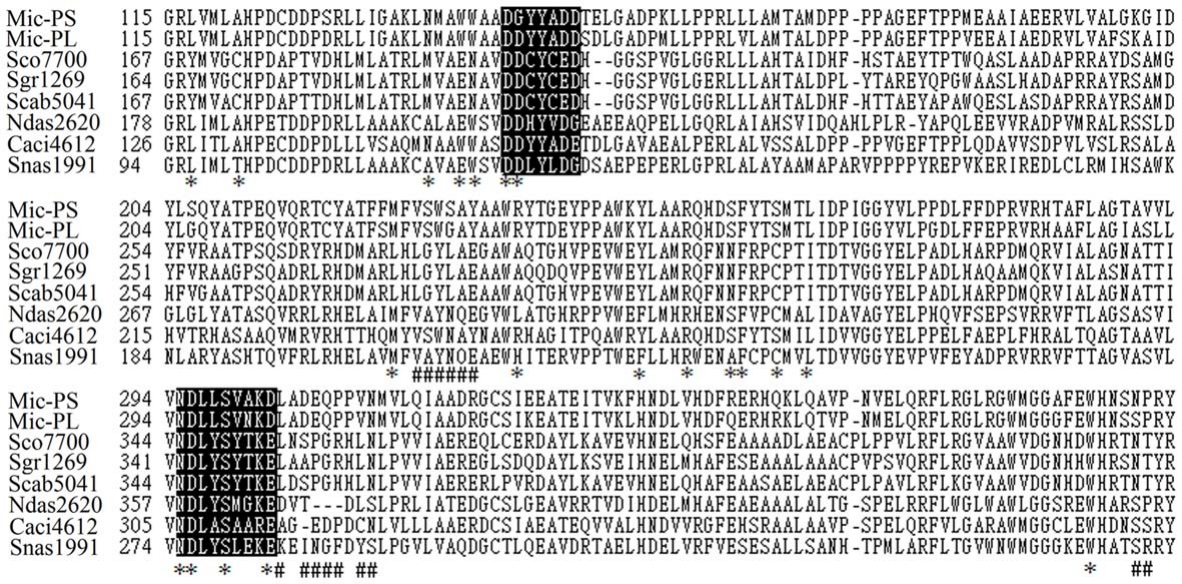
Alignment of amino acid sequences of 2-MIB cyclases. Mic-PS and Mic-PL representing proteins from *Pseudanabaena* sp. dqh15 and *Planktothricoides raciborskii* CHAB 3331 respectively; Sco7700, Sgr1269, Scab5041, Ndas2620, Caci4612, and Snas1991 represent proteins from *Streptomyces coelicolor* A3(2) (AL93912), *S. griseus* NBRC 13350 (AP009493), *S. scabiei* 87.22 (FN554889), *Nocardiopsis dassonvillei* DSM 43111 (CP002040), *Catenulispora acidiphila* DSM 44928 (CP001700), and *Stackebrandtia nassauensis* DSM 44728 (CP001778), respectively. Black boxed residues show the possible Mg^2+^ binding sites; sites marked by * and # at the bottom indicate putative substrate binding pockets and active site lid residues respectively.

The *mic* genes of *Pseudanabaena* sp. dqh15 and *Planktothricoides raciborskii* CHAB 3331 strains were functionally annotated using the CDD database specific hits with Terpene cyclase non-plant C1 in sequence characters. The deduced amino acids sequences of *mic* were aligned with Sco7700, Sgr1269, Scab5041, Ndas2620, Caci4612, Snas1991, and with the reported 2-MIB synthases of *Streptomyces coelicolo*r A3(2), *S. griseus* NBRC 13350, *S. scabiei* 87.22, *Nocardiopsis dassonvillei* DSM 43111, *Catenulispora acidiphila* DSM 44928, and *Stackebrandtia nassauensis* DSM 44728 (Figure 4). In general, these proteins demonstrated relatively high levels of sequence conservation and two strictly conserved Mg^2+^-binding motifs were found in all synthases. In the Mic protein of *Pseudanabaena* sp. dqh15, these two motifs were **DG**YYA**D** and **N**DLL**S**VAK**D**. In *Planktothricoides raciborskii* CHAB 3331, minor differences were found and the two motifs were **DD**YYA**D** and **N**DLL**S**VNK**D.** For 2-MIB synthases from other microorganisms within this alignment, motifs are typically aspartate-rich: **DD**xxx**E** and **N**Dxx**S**xxx**E**. In addition, other important catalytic sites, including active site lid residues and substrate binding pocket sites, were also identified and marked (as # and * in Figure 4, respectively). These sites displayed different characteristics, indicating that relative diversity existed among different taxa with higher conservation within related taxa. The Mics of cyanobacteria were shown to be different from Sco7700, Sgr1269, Scab5041, Ndas2620, and Snas1991 at many functional sites, but was highly homologous to Caci4612, which is a putative 2-MIB synthase of *Catenulispora acidiphila* DSM 44928.

Adjacent to the short upstream (88-bp) segment in *mtf* and the downstream (85-bp) segment in *mic*, two cyclic nucleotide-binding protein genes (*cnb*) with high similarity to each other and with the same transcriptional orientation as *mtf* and *mic* were identified in *Pseudanabaena* sp. dqh15 (Figure 3); one was 1401-bp and the other was 1398-bp. Similarly, one 1401-bp *cnb* gene was identified 94-bp upstream of *mtf* and another 1404-bp homologous gene was identified 9-bp downstream of *mic* in *Planktothricoides raciborskii* CHAB 3331.

### Comparison of 2-MIB synthesis genes in different taxa: evolutionary aspects

**Figure 5:**
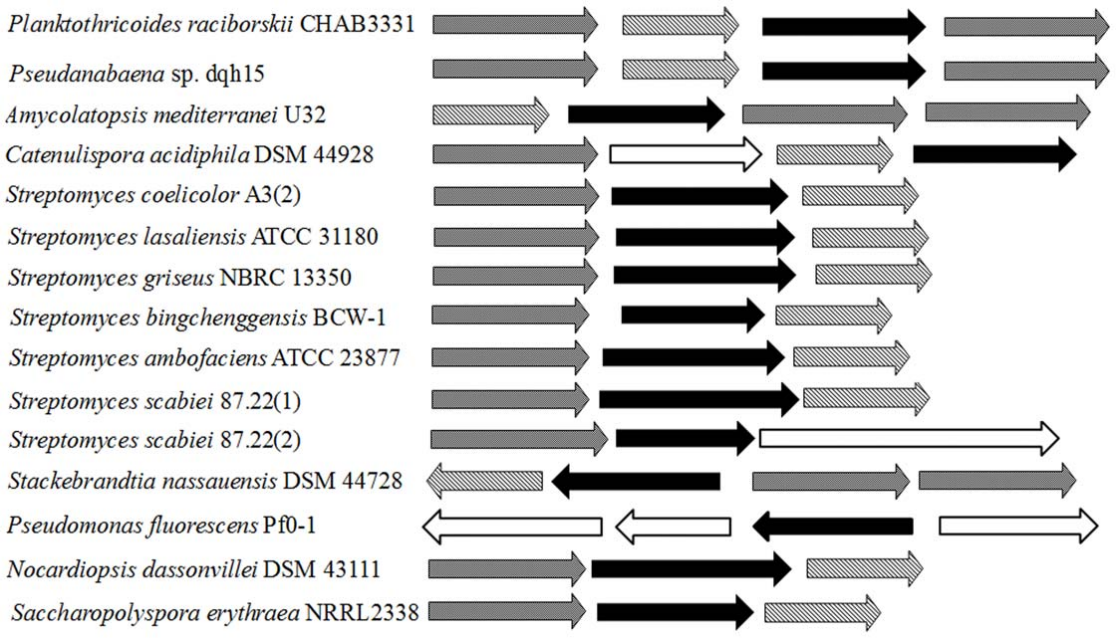
Organization of genes associated with 2-MIB biosynthesis in different organisms. The grayed, oblique-line, filled and opened arrows indicate *cnb*, *mtf*, *mic* and other predicted functional genes respectively.

The 2-MIB synthesis genes and flanking *cnb* genes in the NCBI genome database were identified in 13 bacterial strains using BLAST algorithm. The organization of these genes is shown in Figure 5. Except for *Pseudomonas fluorescens* Pf0-1 and *Streptomyces scabiei* 87.22(2), all strains contained three types of genes: a *mtf* gene, a *mic* gene, and two flanking *cnb* genes. *Pseudomonas fluorescens* Pf0-1 has no putative *mtf* gene and *Streptomyces scabiei* 87.22 lacked a *cnb* gene. The examined strains could be divided into two groups based on the order of genes. In *Pseudanabaena* sp. dqh15, *Planktothricoides raciborskii* CHAB 3331, *Amycolatopsis mediterranei* U32, and *Catenulispora acidiphila* DSM 44928, *mtf* genes were at the front, opposite to the arrangement in most actinomycetes strains. Similarities among all identified *mtf* genes range from 53.5%–90.1% at the DNA level and 37.5%–83.1% at the protein level. For *mic* genes, similarities in DNA sequences ranged from 37.3%–85.4%.

**Figure 6:**
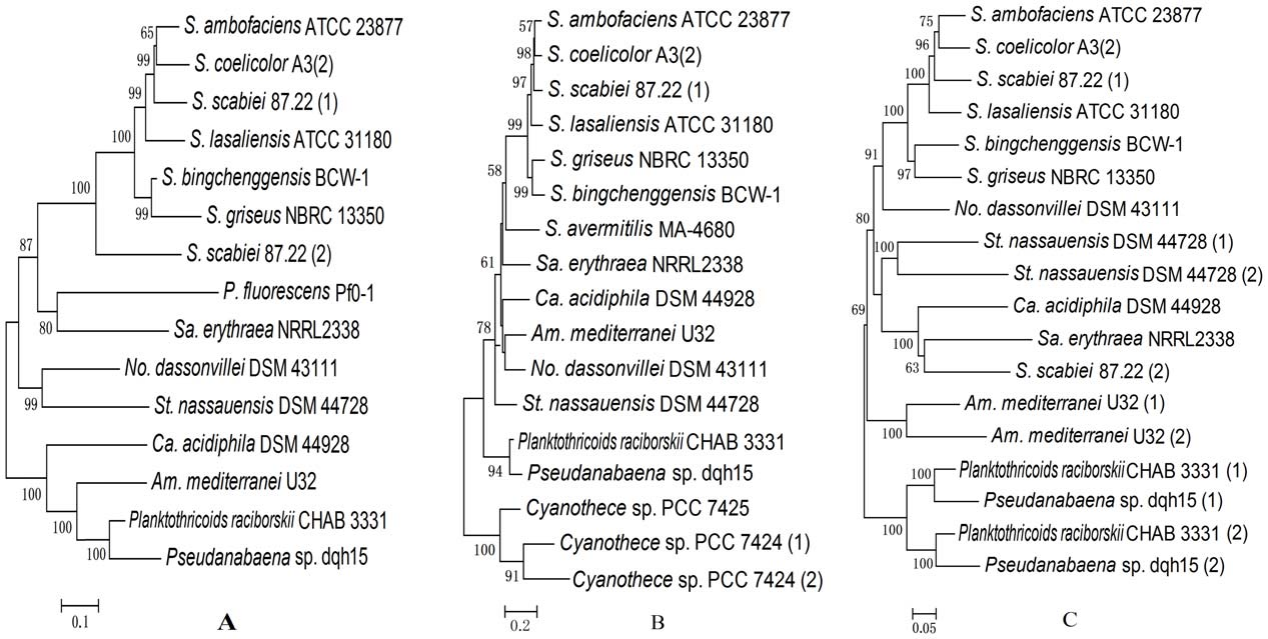
Unrooted neighbor-joining (NJ) phylogenetic trees of 2-MIB associated genes. A: The phylogenetic tree based on putative *mic* genes; B: The tree based on *mtf* genes; C: The tree based on *cnb* genes. Bootstrap values (>50%) are displayed at the nodes. Accession numbers: *Am. mediterranei* U32 (CP002000); *Ca. acidiphila* DSM 44928 (CP001700); *No. dassonvillei* DSM 43111 (CP002040); *P. fluorescens* Pf0-1 (CP000094); *Sa. erythraea* NRRL2338 (AM420293); *St. nassauensis* DSM 44728 (CP001778); *S. ambofaciens* ATCC 23877 (AM238663); *S. bingchenggensis* BCW-1 (CP002047); *S. coelicolor* A3(2) (AL939132); *S. griseus* NBRC 13350 (AP009493); *S. lasaliensis* ATCC 31180 (AB547324); *S. scabiei* 87.22 (FN554889); *Cyanothece* sp. PCC 7424 (CP001291); *Cyanothece* sp. PCC 7425 (CP001344).

An unrooted NJ tree of *mic* genes revealed that the two cyanobacterial strains were grouped together with *Am. mediterranei* U32 and *Ca. acidiphila* DSM 44928, while other actinomycetes and *Pseudomonas fluorescens* Pf0-1 formed another group (Figure 6, A). After searching the cyanobacterial genomes, only three genes from *Cyanothece* sp. PCC 7424 and 7425 were homologous to *mtf*, but the phylogenetic tree based on *mtf* and these three homogenous genes separated *Cyanothece* sp. PCC 7424 and 7425 from 2-MIB-producing strains (Figure 6, B). Similar to the analysis of *mtf* genes, the phylogenetic relationships based on *cnb* genes indicates that the two cyanobacteria strains formed an independent group distinct from the monophyletic clade formed by other actinomycetes (Figure 6, C).

### Expression of 2-MIB synthesis genes in response to light

**Figure 7:**
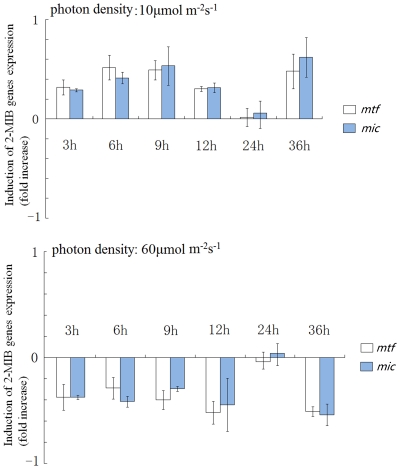
Expression changes of 2-MIB associated genes under different light intensities. The levels of *mtf* and *mic* expression in different light density (10 and 60 µmol photons·m^−2^·s^−1^) were calculated relative to the expression in control culture (30 µmol photons·m^−2^·s^−1^).

The transcriptional response of *mtf* and *mic* genes of *Pseudanabaena* sp. dqh15 under different light intensities was quantified using real-time RT-PCR. Relative expression ratios of 2-MIB-associated genes were obtained and normalized against the16S rRNA. Under low light, there was a 30% increase in the transcription of *mtf* and a 60% increase in *mic* transcription compared to controls within 3–12 h. Under high light intensity, however, transcription of these two genes decreased by 30% (*mtf*) and 50% (*mic*) compared to controls. In addition, after a single 12/12 h light/dark cycle, no apparent transcriptional differences were observed for either *mtf* or *mic* during a subsequent 24 h treatment with low and high light. However, when these cultures were restored to the original conditions after the dark period, the corresponding ∼50% increases or decreases in transcription under low and high light were observed once again (Figure 7, 36 h). Compared with normal light intensity (around 30 µmol photons·m^−2^·s^−1^), the transcriptional behaviors of *mtf* and *mic* increased or decreased quickly within 3 h under low or high lights, and these regulated effects were maintained until the induced factors disappeared.

**Figure 8:**
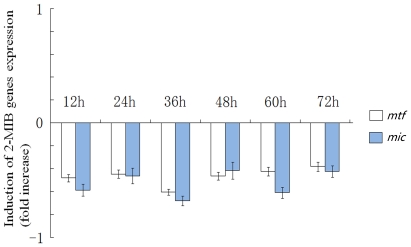
Transcriptional changes in 2-MIB genes under darkness. The levels of *mtf* and *mic* expression under 72 h darkness were calculated relative to the expression in control culture (light density as 30 µmol photons·m^−2^·s^−1^).

Gene expression of *mtf* and *mic* were inhibited when bacteria were cultured in the dark at 25°C for 72 h. Indeed, the respective mRNAs decreased by 40% to 80% compared to controls (Figure 8).

## Discussion

Sporadic episodes of poor-tasting and odorous drinking water have been reported worldwide. Cyanobacteria are a frequent source of these problems due to the production of 2-MIB and geosmin [15], [17], [19]. To date, more than 40 cyanobacterial species have been identified that produce geosmin or 2-MIB, and these species are mainly limited to filamentous groups [3], [5]. In this study, we successfully isolated and verified two cyanobacterial strains from two Chinese lakes, *Pseudanabaena* sp. dqh15 and *Planktothricoides raciborskii* CHAB 3331, that produce 2-MIB. Results imply that cyanobacteria could be the main bio-source of 2-MIB in these lakes and this conclusion is consistent with the HS-SPME and GC-MS results described here. *Pseudanabaena* spp. are common species that have the ability to produce odorous compounds, and many 2-MIB-producing strains have been isolated and studied [3], [20]. However, this study reports one new 2-MIB-producing cyanobacterial taxa, *Planktothricoides raciborskii*, expanding our understanding of the diversity of odor-causing cyanobacteria. Moreover, the two isolated species are water bloom-forming cyanobacteria that could grow massively under suitable environments and cause sporadic cases of poor water quality.

Since they are homologous to the characterized 2-MIB synthesis genes in antinomycetes, the *mtf* and *mic* genes from the two isolated cyanobacteria were suggested to be likely associated with 2-MIB synthesis, although direct evidences from gene mutations or enzymatic experiments are still lacking. Cyanobacteria and actinomycetes should have identical pathways and biochemical mechanisms for 2-MIB synthesis since they possess similar genes. Very recently, Giglio et al. [21] elucidated the genetic and biochemical backgrounds of 2-MIB biosynthesis in another cyanobacterial taxa *Pseudanabaena limnetica* using the method of whole genome sequencing. They reported two adajacent genes including one SAM-dependent methyltransferase gene and another monoterpene cyclase gene, responsible for the conversion of GPP to 2-MIB. It is interesting to find that these two genes are homologous to the corresponding genes illustrated in present study with high identities (more than 90% in DNA sequences), and this finding supports that genes identified in this study are 2-MIB synthesis related. Previous studies have revealed that the two genes associated with 2-MIB biosynthesis in *Streptomyces coelicolar* A3(2) form a two-gene operon and that the gene encoding the cnb protein is located upstream [13], [14]. The intervals between *mtf*, *mic*, and *cnb* were found to be less than 100-bp, suggesting that these genes have no terminal structures and are arranged in the same transcriptional orientation. Therefore, these genes should form an operon. All evidence described above support the idea that the *cnb* gene should be closely related with 2-MIB synthesis. In addition, BLAST and CDD searches indicated that the *cnb* gene(s) was a predicted member of the Crp-Fnr family. The Crp-Fnr family is a universal regulator of photosynthesis and nitrogen fixation in many microorganisms [22]. We speculate that the *cnb* gene is probably involved in the regulation of 2-MIB biosynthesis and physiological responses induced by an increase in intracellular 2-MIB concentration. Geranyl pyrophosphate, the precursor of 2-MIB, is synthesized through the MEP or MVA pathways [23], [24]. Whether *cnb* is also involved in the regulation of these pathways is not known, but warrants investigation.

Compared with typical Mg^2+^-binding motifs (**DD**xxx**E** and **N**Dxx**S**xxx**E**) that are the essential cofactor of all terpenoids [13], [25], the first motif of *Pseudanabaena* sp. dqh15 had a **G** in place of the second **D**, while the last **E** in the NSE triad of both cyanobacterial strains was replaced by a **D**. Combined with the diversity of functional sites, these results suggest that 2-MIB synthases displays many unique features in cyanobacteria. In addition, *Am. mediterranei* U32 and *Ca. acidiphila* DSM 44928 were more closely related to the cyanobacteria strains, as they shared the same arrangement of genes related to 2-MIB synthesis.

The 2-MIB synthesis-associated genes of cyanobacteria were highly homologous to those from actinomycetes, suggesting that 2-MIB biosynthesis (and associated genes) in cyanobacteria and actinomycetes may have a common origin. However, the organization of 2-MIB related genes in cyanobacteria was distinct, indicating that recombinant events may have occurred during evolution. Interestingly, all reported cyanobacteria with the ability to produce 2-MIB are filamentous species belonging to *Oscillatoriales* without heterocyst differentiation [3], [5], [18]. The present study showed that homologous genes with the same genomic arrangement were found in *Pseudanabaena* sp. dqh15 and *Planktothricoides raciborskii* CHAB 3331, and this suggests that all 2-MIB-producing genes in cyanobacteria may have a common origin. Phylogenetic analysis of *mtf* genes demonstrated that cyanobacteria were probably not the origin of 2-MIB-specific methyltransferase genes. Considering the limited distribution of 2-MIB producers among cyanobacteria, it is likely that the capacity for 2-MIB biosynthesis was acquired first by *Oscillatoriales* and then lost in many species during the evolutionary course. The present study also implies that there may be two related evolutionary branches of 2-MIB genes—one represented by actinomycetes and the other by cyanobacteria—and that both share the same origin. Moreover, a putative *mic* gene was also found in *Pseudomonas fluorescens* Pf0-1, a γ-proteobacteria strain, implying that a possible horizontal gene transfer has taken place in many microbial groups. Unfortunately, no studies elucidating the genetic background of these genes in other 2-MIB-producing microbial groups, such as myxobacteria and fungi, have been performed. Evolutionary research on 2-MIB related genes should be conducted in a wide range of microorganisms in the future.

It is well known that environmental factors, such as temperature, light, and nutrient availability, have a great impact on the production of odorous compounds from cyanobacteria [26]–[28]. The precursors of 2-MIB and geosmin, GPP and FPP [4], [11], are also known to serve as intermediates of photosynthetic pigments [29], which suggests that the biosynthesis of these odorous compounds is closely associated with pigment synthesis in cyanobacteria. It has been shown that low light enhanced the synthesis of geosmin in *Anabaena circinalis* and *Lyngbya kuetzingii* [30], [31]. Zimba et al. [29] found that 2-MIB synthesis was regulated by photosynthetic pigments in *Pseudanabaena articulate*, and that the accumulation of lipophilic and phycobilin pigments was positively correlated with 2-MIB accumulation during the early exponential growth phase. Light-induced reactions in the present study indicated that expression of 2-MIB associated genes was inhibited by low light and activated by intense light, consistent with geosmin synthesis as described by Bowmer et al. and Zhang et al. [30], [31]. The increase in the GPP pool concomitant with reduced photosynthesis in low light could activate the transcription of 2-MIB synthesis genes, while GPP depletion under intense light may turn them off. These results suggest that light is a crucial regulator of 2-MIB synthesis. On the other hand, *mtf* and *mic* were still active in the dark (though at lower levels), implying that other internal or external factors activate and regulate the synthesis of 2-MIB in cyanobacteria under darkness. The similar transcriptional responses of both *mtf* and *mic* under changing light conditions confirm that these two genes are located in the same transcriptional and regulatory units. Interestingly, studies on *Phormidium* sp. by Ludwig et al. [10] showed that the expression of putative geosmin genes could not be detected after 24 h in the dark. Whether these results reflect different regulatory mechanisms for 2-MIB and geosmin expression is still unclear and requires further investigation.

## Materials and Methods

### Isolation and cultivation of cyanobacterial strains

Water samples were collected from two eutrophic urban lakes, Dongqianhu in Ningbo City and Donghu in Wuhan City, during taste and odor episodes in 2006 and 2008, respectively. Strains of cyanobacteria were isolated using Pasteur micropipette. Single filaments of cyanobacteria were picked from water samples under the microscope, washed 8–10 times with sterile ddH_2_O, and subsequently placed in a 24-well cell culture plate containing CT medium. After several rounds of purification, strains were finally cultivated in screw-capped glass tubes with CT medium under continuous illumination (30 µmol photons·m^−2^·s^−1^) at 25°C. Two unialgal strains, coded as *Pseudanabaena* sp. dqh15 and *Planktothricoides raciborskii* CHAB3331, were obtained and maintained in the Collection of Harmful Algae Biology Lab, Institute of Hydrobiology of the Chinese Academy of Sciences. Morphological characteristics were examined and cells photographed using an Olympus Model BX51 photomicroscope (Tokyo, Japan).

### Analysis of odorous compounds

Headspace solid phase micro-extraction (HS-SPME) coupled with GC-MS was used to determine odorous compounds based on the procedure of Li et al. [16] with minor modifications. Absorption of odorous compounds was conducted during the exponential growth phase in a 25-ml screw-capped vial containing 5 ml fresh cultivated cells in 5 ml ddH_2_O and 3 g NaCl. The mixture was heated to 60°C and rotated (400 rpm) for 40 minutes. An HSPME fiber (polydimethylsiloxane-divinylbenzene, 65 µm, Supelco 57310-U) was injected into the headspace for absorption.

A gas chromatograph (Hewlett-Packard 6890 plus) equipped with a mass selective detector (Hewlett-Packard Model 5973) was utilized to identify the odorous compounds. After exposure for 40 minutes in the headspace, the HSPME fiber was retracted from the vial and desorpted in the injector (250°C) for 2 minutes in splitless mode. The separation of odorous compounds was conducted on a capillary column (HP-5, Agilent, 0.25 mm × 30 m× 0.25 µm) with the following temperature program: 60°C for 2 min, increased to 200°C at a rate of 5°C per min and held for 2 min, then increased to 250°C at a rate of 20°C per min and held for 2 min. Helium was used as the carrier gas at a constant pressure of 120 KPa. A standard sample of 2-MIB (100 ng· µl^−1^, Supelco, USA) was used to verify the results of GC-MS by comparing retention times and mass spectra.

### DNA extraction, PCR and Genome walking techniques

Genomic DNA was extracted from cyanobacteria cultures by centrifuge (10000 x g, 4°C, 2 min) using a DNA Mini Spin kit (Tiangen, China) according to the manufacturer's instructions. Isolated genomic DNA was dissolved in 50 µl sterile ddH_2_O and stored at −20°C.

The degenerate primers SAMF2 (5′-GAVTTCCTSVTGGRCCACCTCG-3′) and SAMR1 (5′-TCSACGTACATGSTSGACTCGT-3′) based on the sequences of known methyltransferase genes were used to amplify the corresponding homologous fragments of *Pseudanabaena* sp. dqh15 and *Planktothricoides raciborskii* CHAB3331. Amplification by PCR was performed on a Bio-Rad MJ mini personal thermal cycler (MJ Research, USA) in a 20 µl reaction volume containing 0.5U LA Taq DNA polymerase (Takara, Japan), 2 µl 10×PCR reaction buffer, 100 µM dNTP mix, 10 pmol of SAMF2 and SAMR1, and 20–30 ng of genomic DNA. The thermocycle program included denaturation at 94°C for 3 min, followed by 35 cycles of denaturation at 94°C for 30 s, annealing at 55°C for 30 s, extension for 1 min at 72°C, and a final elongation step at 72°C for 5 min.

Based on the amplified methyltransferase gene segments, the genome walking approach was used to amplify their flanking regions. Genome walking-PCR was performed with nested sequence-specific primers (designed on the basis of amplified methyltransferase gene segments) paired with random primers provided in the Genome Walking Kit (Takara, Japan) to amplify the flanking regions using the genomic DNAs as templates. The amplified sequences of upstream and downstream were identified using Blast of NCBI (www.blast.ncbi.nlm.nih.gov/Blast.cgi). Through five rounds of genome walking, four open reading frames (ORFs) with same transcriptional direction were obtained and identified. All PCR and genome walking products were cloned into the PMD18-T vector (Takara, Japan) and sequenced with ABI 3730XL (Invitrogen, USA).

### Light-induced expression of 2-MIB synthesis-associated genes

*Pseudanabaena* sp. dqh15 was selected for experiments on expression of 2 MIB-related genes. Flasks containing 150-ml cultures in late exponential phase (with approximately 1×10^6^ cells/ml) were prepared for investigation of gene regulation under different light intensities. The control was defined as 30 µmol photons·m^−2^·s^−1^, while the low and high light treatments were defined as 10 and 60 µmol photons·m^−2^·s^−1^. All cultures were maintained at 25°C under the 12∶12 h light/dark photoperiod. Samples were collected at 3, 6, 9, 12, 24, and 36 h for genes expression analysis. To test the expression levels of 2-MIB associated genes without light, cultures were cultivated in darkness at the temperature of 25°C for 72 h. Samples were collected every 12 h and treated as described above. Cultures at the same temperature under 30 µmol photons·m^−2^·s^−1^ were used as control. All experiments were performed in triplicate and the results were statistically analyzed by one-way ANOVA to verify significant differences (*p*<0.05).

### RNA extraction, reverse transcription, and real-time RT-PCR

Cyanobacteria cells were harvested by centrifugation (12000 xg, 4°C, 5 min) from 20-ml cultures from each treatment group and resuspended in 1 ml Trizol reagent (Invitrogen, USA). Mixtures were transferred to 2-ml screw-capped vials containing 0.5 ml mini-beads (Biospect, USA). After three beating cycles of 20 s in a mini-beadbeater (Biospect, USA), the lysed cell mixture was frozen at −80°C in preparation for RNA extraction. Total RNA was extracted using the Trizol reagent according to manufacturer's instructions. Finally, total RNA was dissolved in 30 µl RNase-free ddH_2_O and stored at −80°C. RNA integrity was examined by electrophoresis (2% agarose) with ethidium bromide staining (with two predominant ribosomal RNA bands, 28S and 18S) and purity was evaluated by A_260_/A_280_ absorption ratio (1.8–2.0). RNA samples were quantified using spectrophotometry. RNA concentration  =  A_260_ × dilution rate × 40 (ng/L). Samples of RNA were treated with 20 U of RNase-free DNase (Promega, USA) at 37°C for 60 min and then at 65°C for 10 min to remove contaminating genomic DNA, and 0.5 µg of total RNA was reverse-transcribed to cDNA using a reverse transcription kit (Takara, Japan) with 10 µl reaction system containing 1× buffer, 0.5 µl enzyme mix and 200 pmol random 6 mers according to the kit manual. The mixture was incubated at 37°C for 15 min and heated to 85°C for 5 s to stop reaction.

Real-time RT-PCR was used to analyze the expression dynamics of the methltransferase and 2-MIB cyclase genes induced by different light treatments and was performed in a MyiQ mini real-time system (Bio-Rad, USA). The housekeeping gene, 16S rRNA, was selected as the control to normalize the expression of target genes. Primer pairs Mtsf (5′-CGATTGGTCGGTATTAGAGGCT-3′) and Mtsr (5′-ATCACGCGGTCATCAGGCTT-3′), and Mtcf (5′-CGCTCGCTTTGTGAGTGAGATAG-3′) and Mtcr (5′-GGCAGTAGAGTGGTGAGGCAGTT-3′) designed in this study were used to amplify the methltransferase and 2-MIB cyclase genes, respectively. Primers P16f (5′-ACGGAGTTAGCCGATGCTTATTC-3′) and P16r (5′-CGAAAGCCTGACGGAGCAATA-3′) were used for amplification of the 16S rRNA gene. The 20 µl RT-PCR reaction mixture consisted of 0.5 µM of each primer, 10 µl of hot start SYBR GreenI reaction mix (Toyobo, Japan), 8 µl of ddH_2_O, and 1 µl cDNA as template. The cDNA was diluted 1000 times for the amplification of the 16S rRNA gene. The PCR program was set as follows: preheating for 3 min at 94°C, followed by 40 cycles of 94°C for 15 s and 59°C for 15 s. All samples were amplified in triplicate.

The induction ratio was calculated using the formula Ratio  =  2^−ΔΔCt^ where ΔΔCt  =  (Ct, target gene – Ct, 16S rrn) stress – (Ct, target gene – Ct, 16S rrn) control according to the handbook of MyiQ. The results are presented as percentage change of expression.

### Bioinformatics analysis

BLAST algorithm was used to search for sequences homologous with *sco7700* and *sco7701* in the bacterial genome database of NCBI (www.blast.ncbi.nlm.nih.gov/Blast.cgi). All genes homologous with *sco7700* and *sco7701* and their adjacent genes (that are assumed to be in one transcriptional unit) were selected and analyzed. Identification of the ORFs of the amplified sequences was completed by the NCBI ORF finder (www.ncbi.nlm.nih.gov/gorf/gorf.html). For the characterization of 2-MIB cyclase, prediction of functional motifs and sites was performed by another protein functional annotations database, CDD (Conserved Domain Database) (www.ncbi.nlm.nih.gov/Structure/cdd/cdd.shtml) [32].

Unrooted neighbor-joining (NJ) phylogenetic trees based on the methyltransferase gene, 2-MIB cyclase gene, and cyclic nucleotide-binding protein gene were constructed by Mega 4.0 [33] with a bootstrap value of 1000. Sequences of the 2-MIB associated genes in *Pseudanabaena* sp. dqh15 and *Planktothricoides raciborskii* CHAB 3331 strains have been submitted to the NCBI nucleotide database in operon structure under accession numbers HQ830028 and HQ830029, respectively. In addition, the 16S rDNA sequences of these two cyanobacterial strains were also submitted to the NCBI, and their accession numbers are JF429939 for *Pseudanabaena* sp. dqh15 and JF429938 for *Planktothricoides raciborskii* CHAB 3331, respectively.

## References

[1] Gerber NN, Lecheval HA (1965). Geosmin an earthy-smelling substance isolated from actinomycetes.. Appl Microbiol.

[2] Börjesson TS, Stöllman UM, Schnürer JL (1993). Off-odorous compounds produced by molds on oatmeal agar: identification and relation to other growth characteristics.. J Agric Food Chem.

[3] Izaguirre G, Taylor WD (2004). A guide to geosmin- and MIB-producing cyanobacteria in the United States.. Water Sci Technol.

[4] Dickschat JS, Nawrath T, Thiel V, Kunze B, Müller R (2007). Biosynthesis of the off-flavor 2-methylisoborneol by the myxobacterium *Nannocystis exedens*.. Angew Chem Int Ed.

[5] Jüttner F, Watson SB (2007). Biochemical and ecological control of geosmin and 2-methylisoborneol in source waters.. Appl Environ Microbiol.

[6] Medsker LL, Jenkins D, Thomas JF, Koch C (1969). Odorous compounds in natural waters. 2-Exo-hydroxy-2-methylbornane, the major odorous compound produced by several actinomycetes.. Environ Sci Technol.

[7] Wnorowski AU (1992). Tastes and odors in the aquatic environment: a review.. Water SA.

[8] Young WF, Horth H, Crane R, Ogden T, Arnott M (1996). Taste and odour threshold concentrations of potential potable water contaminants.. Wat Res.

[9] Jiang JY, He XF, Cane DE (2007). Biosynthesis of the earthy odorant geosmin by a bifunctional *Streptomyces coelicolor* enzyme.. Nat Chem Biol.

[10] Ludwig F, Medger A, Börnick H, Opitz M, Lang K (2007). Identification and expression analyses of putative sesquiterpene synthase genes in *Phormidium* sp. and prevalence of *geoA*-like genes in a drinking water reservoir.. Appl Environ Microbiol.

[11] Giglio S, Jiang JY, Saint CP, Cane DE, Monis PT (2008). Isolation and characterization of the gene associated with geosmin production in cyanobacteria.. Environ Sci Technol.

[12] Bentley R, Meganathan R (1981). Geosmin and methylisoborneol biosynthesis in streptomycetes: Evidence for an isoprenoid pathway and its absence in non-differentiating isolates.. FEBS Lett.

[13] Komatsu M, Tsuda M, Ōmura S, Oikawa H, Ikeda H (2008). Identification and functional analysis of genes controlling biosynthesis of 2-methylisobornrol.. Proc Natl Acad Sci USA.

[14] Wang MC, Cane DE (2008). Biochemistry and molecular genetics of the biosynthesis of the earthy odorant methylisoborneol in *Streptomyces coelicolor*.. J Am Chem Soc.

[15] Tabachek JAL, Yurkowski M (1976). Isolation and identification of blue-green algae producing muddy odor metabolites, geosmin, and 2-methylisoborneol in saline lakes in Manitoba.. J Fish Res Board Can.

[16] Li L, Wan N, Gan N, Xiao BD, Song LR (2007). Annual dynamics and origins of the odorous compounds in the pilot experimental area of Lake Dianchi, China.. Water Sci Technol.

[17] Graham JL, Loetin KA, Meyer MT, Ziegler AA (2010). Cyanotoxin mixtures and taste-and-odor compounds in cyanobacteria blooms from the midwestern United States.. Environ Sci Techol.

[18] Komàrek J, Anagnostidis K (2005). Cyanoprokaryota 2.. Teil: *Oscillatoriales*. Süsswasserflora von Mitteleuropa 19/2. München: Elsevier GmbH.

[19] King JM, Dew T, Schrader KK, Rimando AM (2003). Catfish off-flavors and their elimination.. Off-Flavors in aquaculture.

[20] Izaguirre G, Taylor WD (1998). A *Pseudanabaena* species from Castaic Lake, California, that produces 2-methylisoborneol.. Wat Res.

[21] Giglio S, Chou WKW, Ikeda H, Cane DE, Monis PT (2011). Biosynthesis of 2-methylisoborneol in cyanobacteria.. Environ Sci Technol.

[22] Korner H, Sofia HJ, Zumft WG (2003). Phylogeny of the bacterial superfamily of Crp-Fnr transcription regulators: exploiting the metabolic spectrum by controlling alternative gene programs.. FEMS Microbiol Rev.

[23] Kuzuyama T (2002). Mevalonate and nonmevalonate pathways for the biosynthesis of isoprene units.. Biosci Biotechnol Biochem.

[24] Spiteller D, Jux A, Piel J, Boland W (2002). Feeding of [5,4-^2^H_2_]-1-desoxy-D-xylulose and [4,4,6,6,6-^2^H_5_]-mevalolactone to a geosmin-producing *Streptomyces* sp. and *Fossombronia pusilla*.. Phytochemistry.

[25] Christianson DW (2006). Structural biology and chemistry of the terpenoid cyclases.. Chem Rev.

[26] Naes H, Post AF (1988). Transient states of geosmin, pigments, carbohydrates and proteins in continuous cultures of *Oscillatoria brevis* induced by changes in nitrogen supply.. Arch Microbiol.

[27] Saadoun IMK, Schrader KK, Blevins WT (2001). Environmental and nutritional factors affecting geosmin synthesis by *Anabaena* sp.. Water Res.

[28] Watson S, Ridal J (2004). Periphyton: a primary source of widespread and severe taste and odour.. Water Sci Technol.

[29] Zimba PV, Dionigi CP, Millie DF (1999). Evaluating the relationship between photopigment synthesis and 2-methylisoborneol accumulation in cyanobacteria.. J Phycol.

[30] Bowmer KH, Padovan A, Oliver RL, Korth W, Garf GG (1992). Physiology of geosmin production by *Anabaena circinalis* isolated from the Murrumbidgee River, Australia.. Water Sci Technol.

[31] Zhang T, Li L, Song L, Chen W (2009). Effects of temperature and light on the growth and geosmin production of *Lyngbya kuetzingii* (Cyanophyta).. J Appl Phycol.

[32] Marchler-Bauer A, Anderson JB, Chitsaz F, Derbyshire MK, Deweese-Scott C (2009). CDD: specific functional annotation with the Conserved Domain Database.. Nucleic Acids Res.

[33] Tamura K, Dudley J, Nei M, Kumar S (2007). MEGA4: Molecular evolutionary Genetics Analysis (MEGA) software version 4.0.. Mol Biol Evol.

